# Metaphor and hyperassociativity: the imagination mechanisms behind emotion assimilation in sleep and dreaming

**DOI:** 10.3389/fpsyg.2015.01132

**Published:** 2015-08-18

**Authors:** Josie E. Malinowski, Caroline L. Horton

**Affiliations:** ^1^Department of Psychology, University of BedfordshireLuton, UK; ^2^Department of Psychology, Bishop Grosseteste UniversityLincoln, UK

**Keywords:** sleep, dreaming, emotion, memory, hyperassociativity, metaphor

## Abstract

In this paper we propose an emotion assimilation function of sleep and dreaming. We offer explanations both for the mechanisms by which waking-life memories are initially selected for processing during sleep, and for the mechanisms by which those memories are subsequently transformed during sleep. We propose that emotions act as a marker for information to be selectively processed during sleep, including consolidation into long term memory structures and integration into pre-existing memory networks; that dreaming reflects these emotion assimilation processes; and that the associations between memory fragments activated during sleep give rise to measureable elements of dream metaphor and hyperassociativity. The latter are a direct reflection, and the phenomenological experience, of emotional memory assimilation processes occurring during sleep. While many theories previously have posited a role for emotion processing and/or emotional memory consolidation during sleep and dreaming, sleep theories often do not take enough account of important dream science data, yet dream research, when conducted systematically and under ideal conditions, can greatly enhance theorizing around the functions of sleep. Similarly, dream theories often fail to consider the implications of sleep-dependent memory research, which can augment our understanding of dream functioning. Here, we offer a synthesized view, taking detailed account of both sleep and dream data and theories. We draw on extensive literature from sleep and dream experiments and theories, including often-overlooked data from dream science which we believe reflects sleep phenomenology, to bring together important ideas and findings from both domains.

## Aims of the Paper

The ways in which our emotions function (or malfunction) in waking life are greatly affected by sleep. For example, sleep benefits the consolidation of emotional memories, and enables us to regulate our emotional reactions (see Emotion-Processing Theories of Sleep). Many theories, of both sleep functioning and of dream functioning, exist to account for the large body of data demonstrating the importance of sleep and dreaming for waking-life emotions. However, often these theories suffer from a lack of integration: theories of sleep do not adequately consider dream research data, and theories of dreaming do not adequately consider sleep research data. Though recently there has been some evidence of a move toward a more integrative approach (e.g., Payne, 2010; Perogamvros et al., 2013; Wamsley, 2013, 2014) sleep and dream theories tend to remain relatively disparate. One of the potential barriers is the skepticism with which dream reports are sometimes viewed; but recently researchers have argued that (a) dream reports are transparent accounts of dream experiencing (Windt, 2013), and (b) dreams are direct reflections of sleep processes (e.g., Payne, 2010; Wamsley, 2013, 2014). As such we argue that greater attention should be paid to dream research and theorizing, as dreams can provide phenomenological accounts of sleep processes.

On the basis of neuroscientific and behavioral data we propose an emotion assimilation function sleep and dreaming, whereby emotions act as a marker for the salience of a memory, thus prioritizing its subsequent consolidation. Emotional memories are then strengthened and assimilated into existing memory schemas during sleep, resulting in memory transformations such as creativity, insight, and gist extraction. An outcome of this process may also be the amelioration of the emotional intensity of the memory. We argue that dreams can be seen to reflect these sleep-dependent mental processes. Our proposed theory attempts to decipher explicitly both one of the mechanisms by which a to-be-remembered item may be selected for subsequent consolidation during sleep (emotional intensity), and further some of the possible mechanisms by which the items may be subsequently assimilated into the wider memory schema, as reflected by dream content (metaphor and hyperassociativity/bizarreness). We review an extensive body of literature and evidence from the sleep and dreaming fields to explore the effects of sleep on emotional functioning. By critiquing current theories we consider what any new approach needs to achieve in order to fit the data across neuroscientific, behavioral, lab-based and home dream recall domains (Desseilles et al., 2011). Our theory is proposed as *a* function of sleep and dreaming, rather than the function of sleep and dreaming: we recognize that there are likely to be many functions of sleep and dreaming, and that the theory likely will not account for all types of dreaming.

In the following sections we: (1) review some emotion-processing theories of sleep; (2) review some emotion-processing theories of dreaming; (3) consider some arguments for and against currently existing emotion-processing theories of dreaming, noting in particular what the existing theories lack; (4) outline our proposal for an emotion assimilation function of sleep and dreaming; and (5) consider two of the mechanisms that we believe underlie emotion assimilation: metaphor generation and hyperassociative thinking. Finally, we suggest some ideas for future directions and draw conclusions about our emotion assimilation theory. Throughout we focus on data from human rather than animal studies and consequently draw conclusions relevant to human functioning.

## Emotion-Processing Theories of Sleep

### Sleep and Emotional Reactivity

Evidence abounds for the importance of sleep for emotional functioning in waking life. For instance, studies have found that sleep benefits emotional reactivity in waking life: sleep deprivation increases reactivity to negative stimuli (Franzen et al., 2009), to anger and fear emotions (Gujar et al., 2010), and indeed also to positive stimuli (Gujar et al., 2011). The latter indicates an overall overreactivity to emotional stimuli following sleep deprivation, suggesting a general modulating effect of sleep on emotions. A review of insomnia and emotions found that insomnia is associated with various mood disorders (e.g., depression and anxiety) and heightened emotional reactivity (Baglioni et al., 2010). Additionally there is compelling evidence that the hormones cortisol and epinephrine contribute to the selective processing of emotional material during sleep (Cahill and Alkire, 2003; Payne and Nadel, 2004), with levels being particularly heightened during REM.

Evidence such as this has led to several theories suggesting that a function of sleep includes emotion regulation or processing. Walker’s (2009) review concluded that REM sleep facilitates the recall of emotional memories, whilst simultaneously reducing the emotional impact. One particularly pertinent study found that the hippocampus (for memory) and the amygdala (for emotion) are both more active when participants are shown emotional compared to neutral stimuli, but when shown the same material months later only the hippocampus is more active (Dolcos et al., 2005), suggesting the memory is strengthened for recall, but emotional reactivity is weakened. Walker hypothesized that this process occurs during sleep, and thus sleep loss results in long-term affective issues, such as chronic anxiety. Walker and van der Helm’s (2009) ‘Sleep to remember, sleep to forget’ hypothesis adds to this theory that if this amelioration of emotions is not achieved on the first night, the process will continue on subsequent nights. Thus, the process occurs in three stages: memory consolidation, memory integration, and emotion amelioration. Further, sleep may prepare the individual for next-day emotional functioning, as proposed by Goldstein and Walker (2014), whose model focused on circadian (i.e., circa 24-h) rhythms.

However, there are also some conflicting results in studies investigating the effect of sleep on mood. One study found that sleep deprivation alleviates depressive symptoms (Vogel et al., 1975), and the same was found also in a study with a non-depressed sample, for whom deprivation was related to emotional adaptation to a negative stimulus (Lara-Carrasco et al., 2009); these findings suggest that it is the *lack* of sleep, rather than the presence of it, that enhances emotion regulation. Vandekerckhove and Cluydts (2010) theory, which draws similar conclusions to those cited above, includes also a suggestion that can account for such disparate findings. The authors suggest that emotional disorders such as depression may be due to an overstimulation of sleep processes (such as greater amygdala activation, higher amounts of REM sleep, and more negative affect in dreams), which results in an excessive emotional response, particularly to negative stimuli. This would explain why sleep deprivation benefits depressed individuals. Although this does not directly explain why sleep deprivation also benefits non-depressed individuals, it may be speculated that during the first night following exposure to a negative stimulus, sleep overstimulation (in response to the stimulus) may result in lack of adaptation, and thus deprivation aids adaptation; but perhaps on following nights the trend would be reversed as sleep adjusts to the stimulus, and then sleep may aid further adaptation. This is in line with Walker’s (2009) idea that the process occurs over multiple nights until adaptation is achieved. A similar model has been espoused in relation to dream incorporation of negative stimuli, the Disruption-Avoidance-Adaptation model (Wright and Koulack, 1987), which will be explored further later, in Section “Wright and Koulack: The Disruption-Avoidence-Adaption Model.”

### Sleep and Emotional Memory Consolidation

Studies have also found that sleep is beneficial for emotional memory consolidation. For instance, recall of emotional stimuli has been shown to be better after sleep than wakefulness (Hu et al., 2006); this is especially true for late-night, rapid-eye-movement- (REM-) rich sleep (Wagner et al., 2004), although recent evidence has demonstrated the complementary roles of both slow-wave sleep and REM (Cairney et al., 2014). Similarly, emotionally related items are recalled more than neutral items following sleep using a false memory task (Horton and Knott, unpublished manuscript). Sleep also ‘unbinds’ waking-life stimuli, selectively consolidating emotional rather than neutral aspects of it, both over the course of a night and during a nap (Payne et al., 2008, 2015).

On the basis of such lines of evidence as these, several researchers have argued that sleep is integral to emotional memory consolidation (e.g., Stickgold et al., 2001; Payne, 2010; Wamsley and Stickgold, 2011). This theory of sleep, thought to be reflected in dreams (Wamsley, 2014), is somewhat divergent from the emotion-processing theories of sleep and dreams, in that instead of waking-life emotional experiences being dreamt of in order to regulate or process emotions, these memory-centered theories focus on the integration of memories. According to these theories the meaning of an experience is extracted, the overall ‘gist’ of the memory rather than specific details are consolidated for recall and use, the emotionally salient rather than the neutral aspects of a memory are strengthened for recall but the emotion itself is dampened, and the memory is ‘unbound’ to enable memories to be reorganized in novel and creative ways, which facilitates creative thinking during wakefulness. Thus, it is a theory of the *assimilation* and reordering of emotional experiences, rather than the regulation or processing *per se*; but assimilation includes the dampening of emotions as a natural corollary of the process, and indeed perhaps is one (of many) of the functions of the process.

Stickgold and Walker (2013), in their ‘triage’ theory argue that it is now “incontrovertible” (p. 139) that a sleep-dependent memory consolidation process exists; but what remains to be discovered is how we decide which memories are selected for consolidation during sleep. They postulate that stimuli are reviewed at the point of encoding, and ‘tagged’ if deemed sufficiently salient and relevant for future use. Three ways in which such information may be tagged are suggested: the emotional intensity of the experience, the future relevance of the experience (e.g., knowing that the information will be useful for a reward or a test later), and the deliberate intention to retain the information. Once the item has been selected for consolidation, two main processes then occur: item integration, in which newly learnt memories are assimilated into pre-existing memory schemas, and multi-item generalization, in which new items are combined into a new schema. We re-visit these ideas in relation to emotional processing in Section “Emotion-Processing Theories of Dreaming” shortly in light of evidence from dream science.

### Mapping Sleep onto Dreams

Sleep is the primary concern of the evidence and theories discussed so far. However, some researchers do reference dream research, with some even mapping emotion-processing theories of sleep onto theories of dreaming (e.g., Walker, 2009; Walker and van der Helm, 2009; Desseilles et al., 2011). However, such speculations often rest on an idea of REM sleep specifically being related to dreaming, since it is usually REM sleep that is implicated; or at least, REM sleep being related to a type of dreaming that is highly emotional, as well as vivid, imagistic, bizarre, complex, and so on (e.g., van der Helm and Walker, 2012). But as many researchers have shown (e.g., Solms, 2000; Domhoff, 2003), REM sleep and dreaming are doubly dissociable, and NREM dreams can take on the typical properties of REM dreams, especially later in the night (Wamsley et al., 2007; Nir and Tononi, 2010; Payne, 2010). Thus, emotion-processing theories of dreaming must look at dream research specifically, rather than extrapolating from REM sleep to dreams (see also Desseilles et al., 2011, for a review of the relationships between brain processes, sleep and dreaming). In the next section we outline some emotion-processing theories that are primarily based on dream research.

## Emotion-Processing Theories of Dreaming

### Hartmann: Dreams “Calm the Storm”

One of the best known theories of emotion-processing in dreaming is that of Hartmann (1996a, 1998, 1999a,b, 2011), who drew conclusions about emotion-processing during dreaming from his work with victims of trauma. He argued that dreams picture waking-life emotions, and that this can be perceived particularly well in nightmares: for example, feelings of terror, vulnerability, or guilt might be pictured in what he termed ‘explanatory metaphors’ in dreams, such as being engulfed by a tidal wave. He suggested that this picturing of waking-life emotions in dreams enables the emotional memory to be connected to other related memories already stored in the dreamer’s mind, in order to reduce the emotional intensity and distress caused by the experience by comparing it to other experiences (“calm the storm”); and second, to prepare the dreamer for any future experiences that might occur. His theory recalls an earlier stress-mastery theory of dreams (Breger, 1967), in which it was suggested that we dream of our stressful waking-life experiences in such a way that the experience is “symbolically blended” (p. 24) with past memories, which enables the eventual mastery of the experience; and both of these earlier dream theories are strikingly similar to Walker and van der Helm’s (2009), discussed in Section “Sleep and Emotional Reactivity.” In all three accounts, at least one of the functions of sleep/dreaming is proposed to be the reduction of the emotional intensity of waking-life experiences.

### Levin and Nielsen: The AMPHAC/AND Model

A similar proposal has been made by Levin and Nielsen (2007, 2009), Nielsen and Lara-Carrasco (2007), Levin et al. (2010), but with the emphasis on the reduction of fear emotions specifically, in their AMPHAC/AND model. According to the model, the dreaming process works in three stages: memory-element activation, in which memory elements (as opposed to intact memories) are activated; memory-element recombination, in which memory-elements are re-organized into a narrative; and emotional expression, which is often fear. As in Hartmann’s and Breger’s theories in the previous section, there is the suggestion that the new (emotional) memories are compared with other memories in order to recombine them into something new. In the AMPHAC/AND model, this is the reason for dream bizarreness: the recombination of memory-elements makes dreams bizarre, because it incorporates disparate elements of memory and blends them together. The authors further suggest that when the process dysfunctions, disturbed dreaming (nightmares and bad dreams) occur, and it can also fail when the memory-elements are not recombined in novel ways but in ways that have already been experienced, which leads to recurrent dreaming. They further note that current situation and individual differences affect how well this process can function; nightmares are more likely when the individual experiences greater ‘affect load’ (amount of stressful/emotional experiences) and ‘affect distress’ (the predisposition toward such experiences being problematic), which vary as a product of situation and personality. These important ideas will be explored later.

### Revonsuo: The Threat Simulation Theory of Dreaming

Another theory that proposes, like Hartmann, that dreaming prepares the dreamer for future experiences is Revonsuo (2000), Valli et al. (2005) Threat Simulation Theory (TST) of dreaming. This is an evolutionary explanation of dreaming, supported by a large amount of data concerning nightmares, the prevalence of fear in dreams, and the repetitive nature of some dream acts. The TST proposes that dreaming provides an opportunity to rehearse possible outcomes that may be useful to survival during wakefulness. One of the central propositions of the TST is that dreams are not an accurate reflection of waking reality, but rather are biased toward threatening experiences. Whilst the disproportionately high presence of negative emotions have been demonstrated in a large sample of dreams (Hall and Van de Castle, 1966), systematically sampled and self-rated dream reports indicate that emotions in dreams are more varied and balanced (Schredl and Doll, 1998; Sikka et al., 2014); however, the TST may still apply to the subset of dreams that represent threat and fear emotions.

### Cartwright: The Emotion Regulation Function of Dreaming

Cartwright (2011), on the basis of her extensive work with divorced individuals (e.g., Cartwright et al., 1984, 2001, 2006; Cartwright, 1991), also proposed an emotion-regulation function of dreaming. She proposed that if we experience negative waking-life experiences and dream of those experiences in specific ways, such as with negative emotions and with time variance (dreams set in the past, present, and future), then we are more likely to show improvement on coping with those experiences than if we do not dream of them or dream of them in the wrong way, such as with neutral emotions or without time variance (dreams set exclusively in the past). Similarly, studies with alcoholics have indicated that dreaming of alcoholism, particularly in negative tones, is related to recovery (Choi, 1973; Kibira, 1995; Parker and Alford, 2010). Although Lara-Carrasco et al.’s (2009) study (see Sleep and Emotional Reactivity) suggested a link between *lack* of emotional adaptation and the appearance of negative emotions in dreams, it may be that this difference occurred because of the different methodologies: Cartwright’s studies measured naturalistic stimuli over long time periods, whereas Lara-Carrasco et al. (2009) used a stimulus-manipulation paradigm over a single night. It may be that the emotion-regulation function is only effective for naturalistic, salient, and/or long-lasting waking-life experiences such as divorce. This is in line with Walker’s (2009) assertion that emotion amelioration may not be achieved on the first night but may require several nights of processing: it may be that incorporation *initially* has detrimental effects on adaptation, but, longer-term, incorporation is beneficial. This is what is proposed by the next model by Wright and Koulack (1987).

### Wright and Koulack: The Disruption-Avoidance-Adaptation Model

Wright and Koulack’s (1987) model was designed to account for apparently contradictory findings relating to dream incorporation of emotional waking-life experiences and adaptation to those experiences. In the Disruption-Avoidance-Adaptation model, it is suggested that incorporation (disruption), non-incorporation (avoidance), and finally adaptation are all points along a dream adaptation continuum. First, a stressor is experienced in waking life, and this disrupts sleep and so is incorporated into a dream. This is not necessarily adaptive, in line with the findings that incorporation on the first night after exposure to a negative stimulus is related to maladaptation (Lara-Carrasco et al., 2009), but is a point along a continuum that may *eventually* lead to adaptation. Secondly, the dream avoids that material or complements it in some way, for the purposes of re-finding emotional homeostasis. Disruption/incorporation and avoidance/non-incorporation may then oscillate for as long as necessary until the material is mastered. This last point puts the model on similar ground to theories noted earlier that suggest the process is long term and iterative. It may be that this model is related to the findings regarding the ‘dream-lag effect,’ which shows that waking-life experiences – particularly personally significant ones – are incorporated into dreams on the first night after having been experienced, and then again around 5–7 days later, and the second time they appear they are more abstract (Nielsen and Powell, 1992; Blagrove et al., 2011b; van Rijn et al., 2015). These periods of incorporation, non-incorporation, and re-incorporation with more abstraction may reflect the disruption-avoidance-adaptation process postulated by Wright and Koulack (1987) and – potentially – the processes of memory stabilization, elaboration and consolidation as proposed by Walker (2005). Wright and Koulack (1987) further note that this process only occurs for stressors that are potent enough not to be able to be dealt with by the waking brain alone; benign stressors may be mastered during wakefulness and so do not need incorporation into dreams for mastery to occur there.

### Convergences between the Theories

There are some clear similarities between the theories, both of sleep and of dreaming: emotion-processing during sleep/dreaming is a long-term, iterative process involving both incorporation and non-incorporation of emotional material, and the process involves the stages of memory activation during sleep and dreaming, recombination of those memories (associatively with other memories and with imagination), and finally emotional adaptation. In the next section we consider a number of arguments in favor of and against the emotion-processing theories outlined here. The arguments in favor of the theories that come from sleep research have been noted in Section “Emotion-Processing Theories of Sleep” and will not be repeated here.

## Arguments for and Against Emotion-Processing Theories

### Arguments for Emotion-Processing Theories of Dreaming

#### We Preferentially Dream of Emotional Waking-Life Experiences

As a starting point, it is necessary for any emotion-processing theory of dreaming to show that we preferentially dream of our emotional waking-life experiences over neutral experiences, since if we dream of emotional and neutral waking-life experiences equally it would be more difficult to argue that dreams serve a specific emotion-processing function. Several studies have demonstrated the tendency for dreams to reflect emotional experiences from waking life (Schredl, 2006; Horton et al., 2011; Horton, 2012; Malinowski and Horton, 2014a), and a wealth of content analysis studies indicate that dreams generally reflect waking-life concerns (e.g., Domhoff, 2003). Whilst this could be accounted for by a recall bias, systematically sampled dream reports still show this trend (Schredl and Doll, 1998). Moreover, waking-life experiences that are incorporated into dreams are more emotional than those that are not incorporated (Schredl, 2006; Malinowski and Horton, 2014a). Thus, it can be said quite conclusively that waking-life emotions tend to be incorporated into dreams, and with more frequency than waking-life experiences that are less emotional.

#### Emotional Intensity is a Marker for Recall

Since we know that dreams preferentially incorporate emotional experiences, it then follows to query what purpose emotionality may serve. It is known that emotion is important for memory recall: for example, memories that are emotional are even easier to recall than memories that have been specifically attempted to be remembered (Heuer and Reisberg, 1990). Similarly, emotional memories (regardless of valence) are recalled better than neutral ones (although there are differential effects for negative and positive emotions: Kensinger, 2009). Walker (2009) notes that emotional memories are retained, though the emotion itself is reduced over time. The reason for this may be that it is useful to have an emotional reaction to a stimulus initially in order to gage which experiences should be recalled and which forgotten, but not to retain those emotional reactions alongside the memory. That is, emotions are a useful signifier for tagging memories to be remembered – for example, to avoid negative experiences and repeat positive ones – but it would be detrimental to keep experiencing the emotions each time the memory is activated. Thus, emotional reactions to experiences are an instant, automatic way of alerting ourselves to the importance of those experiences. This aligns with Stickgold and Walker’s (2013) triage theory, in which they suggest that salient information is ‘tagged’ for consolidation by emotional arousal, future relevance, and/or deliberate intention.

Further evidence for this claim comes from the Fading Affect Bias (FAB; Walker and Skowronski, 2009; Ritchie et al., 2014), whereby the negative emotional tone of an autobiographical memory fades faster over time than the positive emotion. This has been evidenced to as quickly as 12 h following the original experience (Gibbons et al., 2011), possibly emerging following a period of sleep. The FAB has been proposed to reflect positive coping, overcoming past experiences, and recalling events in line with the current conceptions of the self. In addition, as we outline later, the heightened emotionality associated with a memory may signify its importance and need for consolidation, for ease of subsequent retrieval, with the emotionality being ameliorated once that purpose has been achieved. The FAB has been shown to occur in memories for dreams (Ritchie and Skowronski, 2008), although it remains to be seen whether dreaming, or sleep, actively contributes to the effect.

#### Dreaming of Emotional Waking-Life Experiences is Related to Subsequent Adaptation

Next, any emotion-processing theory of dreams needs to be able to demonstrate that dreaming of emotional waking-life experiences results in improved adaptation to them compared to non-incorporation. This has been demonstrated in Cartwright’s laboratory as discussed in Section “Cartwright: The Emotion Regulation Function of Dreaming.” She has also studied levels of depression in non-depressed students pre-sleep and post-sleep, and linked this with dream content (Cartwright et al., 1998). Students with moderate scores on the Profile of Mood State (POMS) scale for depression had three time as many negative dreams at the start of the night than toward the end and exhibited a significant reduction in depression scores from night to morning, whereas students with low scores had equal numbers of positive and negative dreams and no alteration in scores from night to morning. This accords with her finding that among depressed divorcees, participants who had more negative dreams at the beginning and fewer at the end were more likely to have been in remission at follow-up 1 year later than those who had more negative dreams and the end and fewer at the beginning of the night. Thus, in addition to the studies discussed earlier, it has been shown that progressive dreaming across the night (negative to positive) is related to improved mood, both short-term and long-term, in both a depressed and a non-depressed sample.

#### Summary of the Evidence for Emotion-Processing Theories of Dreams

Thus these pieces of evidence show that: (1) dreams preferentially incorporate emotional experiences; (2) emotional experiences are more likely to be recalled than neutral ones; (3) sleep may facilitate the amelioration of emotional experiences; and (4) there is a relationship between incorporating emotional experiences in dreams and adapting to those experiences. Taken together, they provide evidence for emotion-processing theories of sleep and dreaming, illustrating that emotionality likely ‘tags’ a memory for recall, and thus such memories appear later in dreams, perhaps for the function of ameliorating and/or adapting to those experiences.

### Arguments Against the Emotion-Processing Theories

#### The Evidence is Correlational, Not Experimental

It has been argued that since Cartwright’s studies were correlation rather than experimental (the stimuli were not manipulated), causality cannot be inferred (Blagrove, 1992, 2011): it might be that, rather than specific dreams resulting in coping ability, coping ability may lead to specific dreams. For example, well-adapted divorcees might be capable of accepting the negativity of their situation and thus dream negatively, whereas divorcees who do not adapt well may be stuck in the past happiness of their marriage and thus have dreams that are set in the past. Thus, the dreams may more simply be a carry-over of waking-life coping mechanisms rather than a coping mechanism outside of wakeful cognition.

More generally, any relationship between emotional processing and dreaming of emotional material needs to be subject to systematic comparisons of dreaming versus not-dreaming. Some theories of dreaming, such as the TST, Hartmann’s “calm the storm” and Cartwright’s “emotion regulation function” theories, emphasize the role of dreaming, as opposed to sleep *per se*, in the processing of emotions. Whilst the other theories use dreams to evidence the processing of emotion during sleep, they typically do not assume that dreaming is anything more than a reflection of oﬄine processing during sleep. At present the methods for sampling dreams both in and out of the laboratory are not yet sophisticated enough to make such a distinction between sleep with dreaming and sleep without dreaming, with dream recall being the only means by which researchers can find evidence of having dreamt (Kahan and Horton, 2012). As such, until effects of dreaming can be experimentally manipulated, we must draw tentative conclusions about any function of dreaming as distinct from a function of having slept. However, some studies have begun to develop methods for matching improvements in specific autobiographical memories with dreams of those precise experiences, noting that emotional experiences are more likely to feature in dreams than neutral ones and that those experiences are preferentially recalled more accurately 2 weeks later (Horton, 2012).

#### Dream Theories Focus on Different Levels of Inquiry to Sleep Theories

Following on from this view, any theory of emotion processing needs to be able to account for the behavioral (as well as the neuroscientific) profile of emotion processing in sleep (as summarized in Section “Emotion-Processing Theories of Sleep”), as well as in dreaming. At present the theories of emotional processing in dreams can, in part, explain the preferential processing of emotional memory (compared to emotionally neutral memories), but they cannot be mapped onto the trends concerning the preferential consolidation of emotional memory following sleep (aside from Horton, 2012, as noted above). Wamsley’s work exploring the experience of dreaming alongside memory processing in sleep (Wamsley et al., 2010; Wamsley, 2014) has been exemplary in attempting to link these domains, though vast methodological improvements are required before conclusions can be confidently drawn concerning the specific role of dreaming in emotional processing during sleep.

#### Valence does not Influence Incorporation

The theories of emotion-processing in Section “Emotion-Processing Theories of Dreaming” focus specifically on negative emotions: Cartwright’s (2011) theory came from work with individuals going through divorce, Hartmann’s (1996a) theory was developed from work with trauma victims, Levin and Nielsen (2007, 2009) focus specifically on fear extinction, and Wright and Koulack (1987) on stressful experiences. Thus, these accounts of dream functioning would suggest that dreams particularly incorporate particularly negatively emotional, and/or stressful waking-life experiences. However, this is not the case. Whilst there is an array of literature concerning negative emotions in dreams, the evidence from dream science suggests that experiences typically incorporated into dreams are more emotionally *intense* than those that are not incorporated, but are not specifically negatively valenced (Schredl and Doll, 1998; Schredl, 2006; Horton et al., 2011; Malinowski and Horton, 2014a). Some studies indicate that there is a ‘negativity bias’ in dreams – the tendency for dreams to be more negatively than positively valenced (Hall and Van de Castle, 1966; Snyder, 1970; Nielsen et al., 1991; Domhoff, 1996) – but others have found a balance between positive and negative emotions in dreams (Strauch and Meier, 1996; Kahn and Hobson, 2002; St-Onge et al., 2005). Furthermore, studies that have compared external ratings of emotions to participants’ self-ratings of emotions find that external raters overestimate negative emotions in dreams compared to what the dreamers themselves perceive in the dreams (Schredl and Doll, 1998; Sikka et al., 2014), indicating that it is the method that produces the negativity bias. In other studies, the top self-reported emotion has been some form of joy, elation, or excitement (Howe and Blick, 1983; Fosse et al., 2001).

In a similar vein, as reported in Section “Emotion-Processing Theories of Sleep,” sleep deprivation affects not only negative waking-life emotional reactivity, but positive reactivity too (Gujar et al., 2011); the authors proposed that sleep loss not only has detrimental effects on the processing of negative stimuli, but has bi-directional effects on emotions. Thus, following this line of evidence, it may be that rather than just enabling adaptation to negative stimuli, sleep more generally facilitates lower levels of reactivity to emotional stimuli. Similarly Carr and Nielsen (2015) have recently found that participants scored higher on a primed associational breadth task following REM but not NREM sleep nor a period of wakefulness, and, moreover, the effect was stronger for positive over negative stimuli, again demonstrating that positively valenced experiences need to be taken into consideration when considering the function of sleep or dreaming on waking life emotions.

Taking the discussion back to dreams, it may be said then that incorporating emotional experiences into dreams is either a reflection of or is itself instrumental in this process, through the assimilation of emotional experiences into the memory system (though the latter view is particularly challenging to evidence). The detrimental effects of sleep loss and/or lack of dream incorporation of emotional stimuli may be more obvious, more pronounced, and/or of more clinical relevance for negative stimuli, but the evidence for positive emotions necessitates a consideration not only of adaptation to negative experiences, but also a more general discussion of assimilation and amelioration of all emotional experiences in sleep and dreams. This is what has been achieved in sleep research theories (see Emotion-Processing Theories of Sleep), whereby both positive and negative emotions are accounted for: dream research theories would thus also benefit from a consideration of such data and theories as these.

#### Emotions vs. Stress

Just as there is a lack of evidence for negatively emotional waking-life experiences being preferentially incorporated into dreams over positive ones, so there is a lack of evidence for stressful experiences over non-stressful ones, whereby stressfulness is conceived of as a specifically negative and aversive longer-term response state (Kasl, 1995), as opposed to emotion which can be negative or positive and which is typically shorter-lived. Furthermore an emotion may elicit an associated subjective feeling, and one such recognized feeling is that of stress. This lack of evidence argues further against some of the dream theories, which specifically discuss stressful waking-life experiences (Breger, 1967; Wright and Koulack, 1987). While there is some evidence that particularly unusual stressful experiences such as trauma are dreamt of (see Barrett, 1996; Punamäki, 2007, for reviews), studies that have measured the effect of more usual stressful experiences on dreams have not found the same pattern. Malinowski and Horton (2014a) measured the emotionality and stressfulness of waking-life experiences separately, and found that experiences that were incorporated into dreams were more emotional, but no more stressful, than those that were not incorporated. Similarly, Delorme et al. (2002) found no effect of examinations on the dreams of students, whether the dreams were measured for direct effect or indirect effect (such as levels of stress or threat emotions in dreams), even though wakeful stress was higher during the examination period than a control period, demonstrating again that stressful experiences do not necessary result in dream incorporation.

Wright and Koulack’s (1987) model could argue that examinations are relatively benign and can be adapted to during wakefulness, and so can account for this discrepancy. Indeed, some of the other results in Delorme et al. (2002) support this theory. When looking at students’ coping mechanisms for the examination period, students who used problem-solving in waking life did not have incorporations of the examinations, whereas using positive reappraisal in waking life – making the problem seem less distressing without solving it – correlated with problem-solving in dreams. The inference is that incorporation of a relatively non-stressful experience becomes necessary if it is not adequately dealt with in waking life. Thus, in this view, it is not merely the level of stress intensity that determines whether or not a stressor needs to be incorporated into dreaming in order to be processed, but also ability to cope with the stressor, highlighting a role for individual differences. This will be explored further in the following section.

#### Individual Differences

For some individuals, dreaming of negative waking-life experiences may actually have the opposite effect to mastering that material, in that it may result in an increase in distress, rather than a decrease. This is in line with Levin and Nielsen’s (2007, 2009) assertion that ‘affect distress’ (the predisposition to be distressed by difficult experiences) mediates whether the dreaming process is to function or dysfunction (become a nightmare or bad dream). Though Levin and Nielsen do account for this factor, many theories do not. Research has shown that nightmare distress (the amount of distress an individual feels about having a nightmare) is more strongly related to well-being than nightmare frequency (Belicki, 1992; Blagrove et al., 2004). Thus, for some individuals, having highly emotional experiences leads to dreaming of the experience – and they may also be predisposed toward such dreams becoming nightmares – and those dreams/nightmares in turn result in more waking-life distress. We have found individual differences that support this contention: individuals who score highly on neuroticism, indicating trait emotional lability, also score highly on a scale that measures how often their waking-life emotions affect their dream emotions and vice versa (Malinowski and Horton, unpublished manuscript).

Waking-life stress has also been shown to relate to experiencing recurrent dreams: active recurrent-dreamers (but not past- or non-recurrent dreamers) had more recurrent dreams in a period leading up to examinations than after (Duke and Davidson, 2002). If recurrent dreams are a malfunction of the ‘normal’ assimilation process of dreaming whereby dream material stagnates and repeats rather than progressing and processing (as suggested by Levin and Nielsen, 2009), then this would indicate that for some individuals waking-life stress results in a malfunction of the dream process (i.e., an increase in recurrent dreams). Being an active-recurrent dreamer has also been shown to be related to a host of waking-life issues summarily described as low levels of ‘psychological well-being,’ a measure that includes levels of waking-life depression, stress, and anxiety (Brown and Donderi, 1986). This research strengthens the argument that individual differences must be taken into account in considering dream function.

Individual differences may also account for Cartwright’s findings: individuals who were predisposed toward coping with their divorce are more likely to have certain types of dreams (such as with time variance), while those predisposed toward failing to cope are more likely to have other types (such as stuck in the past). Indeed, earlier research found that participants who were high in ‘neuroticism’ had more dreams set in the past than in the present (Cohen and Cox, 1975). So it may be that predispositions determine what kind of dream a person will have, rather than the dream determining a person’s ability to adapt.

#### Situational Differences

In addition to individual differences, situational differences affect whether incorporation of highly emotional experiences occurs, or whether non-incorporation follows, and the extent to which this relates to adaptation. In Levin and Nielsen’s (2007, 2009) model this is called affect load (amount of stressful or emotional experiences an individual is experiencing). For example, though some evidence such as Cartwright’s indicated a potentially beneficial role for incorporation, Lavie and Kaminer (1996), who worked with victims of trauma, found that adaptation was not related to dream incorporation of the trauma but to repression of it (and an overall repressive coping style). Thus it may be that higher levels of stress require a repressive coping style, which includes dream repression, whereas lower levels of stress may be better served by focusing on the experiences and mastering them, and therefore includes dream incorporation. This would suggest a modification to Wright and Koulack to the effect that in addition to benign stressors resulting in non-incorporation and potent stressors resulting in incorporation, *extreme* potent stressors may also result in non-incorporation: which in this case is synonymous with repression. As Walker (2009) notes, post-traumatic stress disorder (PTSD) may constitute a malfunction of the decoupling of memory and emotion process; in which case, repression may be the only viable coping mechanism. Punamäki (2007) notes that dream recall may either increase or decrease after traumatic experiences, perhaps again illustrating that different methods of coping may be used by different people according to the situation and individual.

Similarly, studies have shown that the content of traumatic dreams varies depending on the type of trauma experienced and the stage of recovery. Regarding the type of trauma experienced, it has been found that traumatized individuals with direct military experience of war have more direct, unambiguous, and replicative dreams of their experiences, while traumatized civilians have more indirect, ‘symbolic,’ and non-replicative incorporations (Schreuder et al., 1998, 2000). Thus, stressors of all levels of intensity may lead to incorporation but those with lower levels may be harder to detect because they are symbolic. Regarding the stage of recovery, Hartmann (1996b), Wilmer (1996), both working with Vietnam veterans, found that there were three types of traumatic dreams experienced by these individuals: apparently veridical replays of war experiences; variable nightmares that pictured plausible scenarios that did not actually occur; and hallucinatory nightmares that were like ‘ordinary’ nightmares, including material from the previous day. These three types of nightmares were believed to be sequential and indicated stage of recovery: as the trauma of war was adapted to, the dreams become less veridical and more like ordinary dreams (that is, bizarre and symbolic). These studies again show that dream incorporation of, and adaptation to, highly stressful experiences is dependent on various factors, including type of stress and stage of recovery. Hartmann’s and Wilmer’s suggestion that dreams are repetitive but become more bizarre as recovery is made may be empirical evidence for the aforementioned assertion of many of the theories that the process is long-term and iterative.

While many of the emotion-processing theories suggest, with Walker (2009), that PTSD dreaming is a malfunction of the ‘normal’ dreaming process (such as Hartmann, 1996b) and account for the change in dreams and adaptation to the trauma by postulating the dreamwork as a long-term, iterative process, they do not attempt to explain what it is that has malfunctioned: why dreams become repetitive and literal, and therefore unable to serve their emotional memory-assimilation process. We address this issue in Section “The Emotion Assimilation Theory of Sleep and Dreaming,” where we suggest that PTSD dreams are a malfunction of the ‘normal’ dreaming process due to the stunting of imagination, which is the driving force of the quality of dreams (such as bizarre, hyperassociative, and metaphorical) required for ‘normal’ dreaming, which involves the assimilation of emotional memories.

Taken together, the existing theories of emotion processing in both sleep and dreaming share the view that emotion acts as a marker of the importance of an experience, signifying the need for further processing. The reasons for such processing and the mechanisms underlying it have been disputed. We next attempt to synthesize the evidence from both the sleep- and dream-processing fields, in proposing a new theory of emotional memory assimilation during sleep, as evidenced by dream phenomenology.

## The Emotion Assimilation Theory of Sleep and Dreaming

In line with sleep and memory researchers (e.g., Payne, 2010; Wamsley and Stickgold, 2011), we propose that emotional waking-life memories are preferentially activated during sleep, thus appearing in dreams, in order to assimilate these memories into the wider memory system. This serves several purposes: (i) consolidation, which is the strengthening and stabilization of the memory (with emotional memories preferentially selected); (ii) assimilation into the memory system, which is the integration of new memories with past memories, expectations for the future, and imagination; (iii) the creation of abstractions and generalizations from the memory; (iv) the generation of creativity, insight, novel ideas, and also problem-solving; and (v) emotion amelioration.

The process differs depending on the type of situation, the stage in which the process is in, and according to individual differences. Adaptation is something that *may* occur as a product of incorporation and assimilation, but this will vary depending on trait differences, the type of waking-life experience, the intensity of the associated emotionality, the wider situation the individual is currently in, their history, the content of the dream, and probably many other factors. Thus, though some kinds of incorporation have been shown to relate to adaptation, in some individuals such as those who score highly on the personality trait ‘neuroticism,’ incorporation may lead to further emotional unbalance rather than adaptation such that waking-life emotions affect dream emotions and dream emotions in turn affect waking mood. Similarly in some circumstances, such as trauma, extreme emotions can result in non-incorporation (repression), which is, in this situation, necessary for coping. Additionally incorporation may only relate to adaptation when dream content contains specific properties, such as time variance instead of being set in the past.

We propose that the process of assimilating emotional waking-life experiences during sleep and dreaming into pre-existing networks and schemas requires imaginational abilities: for sleep and dreaming to achieve this function, individuals must be able to conjure experiences appearing in dreams during sleep in specific ways, as will be explained in the next section. In the immediate term, information that has been encountered during the day needs to be assessed in terms of its importance and potential future use. The vast amount of stimuli perceived and experienced renders this process somewhat arduous, so emotional information and experiences are first and foremost processed. These emotional experiences are activated during sleep, giving rise to a predictable pattern of dream behaviors. Over time, the emotional stimuli are re-activated in slightly different contexts, as part of the assimilation process. This means that they are better remembered in the short term, the associated emotional intensity is reduced over time during assimilation, and the salient information from the experience is embedded into stable networks for effective and rapid retrieval when needed.

For this to happen, information, and experiences need to be activated repeatedly and in a range of manifestations (Lewis and Durrant, 2011; Horton and Malinowski, 2015). We outline the methods by which this occurs, via dreaming, for emotional material in particular. The clearest way of perceiving how this functions in healthy individuals is to look to the malfunction of the ‘normal’ process: that is, to look at dreams following traumatic experiences. This follows Hartmann’s (1999b) argument that the nightmare is the best type of dream to look at when contemplating dream functioning. As discussed earlier, PTSD dreaming involves the literal replaying of the traumatic experience(s) during sleep, and the literalness of the replaying decreases as adaptation increases. Concomitantly the bizarreness and symbolism, and the associativity with other waking-life experiences, increase also. Underlying these abilities to create bizarre scenarios and metaphors and to make associations between disparate memories is the ability to imagine and to create novel thoughts, and this is the crux of the process. When a person experiences a trauma, their ability to imagine is stunted: not only are PSTD dreams literal rather than bizarre and symbolic, but waking-life imaginational abilities are diminished as well, such as the appearance of more general, and less specific, autobiographical memories (Moore and Zoellner, 2007), including when for when imagining the future (Brown et al., 2012). Furthermore, therapeutic treatments that aim to kick-start imaginational abilities such as Image Rehearsal Therapy (e.g., Krakow et al., 2001) and Focusing-Oriented Dreamwork (Ellis, 2013, 2014) have been shown to reduce PSTD nightmare occurrence and PTSD symptoms.

The more usual (non-clinical) types of repetitively dreaming of the same concern – recurrent dreams and repetitive dream themes – indicate a similar but less heightened malfunction of the ‘normal’ dreaming process; i.e., the experience is not adequately assimilated into the memory system and so is activated over and over again until assimilation is achieved (e.g., Domhoff, 1993; Zadra, 1996; Hartmann, 1998). This is the view also of Levin and Nielsen (2007, 2009) who argue that recurrent dreams are an example of disturbed dreaming, and are a malfunction of the healthy dreaming process. PTSD nightmares and recurrent dreams are variously perceived by researchers either to be an extreme version of normal dreaming (e.g., Domhoff, 1993), or a failure of the normal dream process (e.g., Hartmann, 1998; Levin and Nielsen, 2007, 2009); however, if the trajectory of PTSD dreams is considered, it can be seen that both of these positions are possible: initially the PTSD dream is a malfunction of the normal dream process in that it repetitively fails to be assimilated, but as the trauma is adapted to the dreams change to become more like ‘normal’ dreaming, and so in this sense can be seen as being at the extreme end of the spectrum of dreaming, and that end-of-the-spectrum is synonymous with a failure of the process.

The imagination abilities that are required for ‘normal’ dreaming and which are greatly reduced following trauma, include (but likely are not limited to) the ability to generate metaphors, and the ability to make wide associations between disparate memories (hyperassociativity), which, during sleep and dreaming, are lived, immersive experiences engaging the whole body. These two aspects of imagination are evidenced in dreaming, and, moreover, there is evidence that these kinds of thinking during sleep are associated with the type of creative thinking required to problem-solve and adapt to experiences. In the following section we consider each of these imaginational abilities in turn.

### Metaphors

Metaphors (“statements of non-literal reality”: Tourangeau and Rips (1991, p. 453) for waking life in dreams are often acknowledged to exist but rarely studied in dream science owing to the extreme methodological difficulties in broaching the subject (e.g., Bulkeley and Kahan, 2008). Nevertheless, many of the world’s leading researchers historically and currently subscribe to the notion that dreams can be metaphors for waking life, picturing waking-life experiences and emotions in non-literal, figurative ways (e.g., Jung, 1948a,b; Lakoff, 1993; Hartmann, 1996a; Domhoff, 2003). Some common agreements between researchers exist, including: dream metaphors picture abstract concepts in concrete terms; these metaphors are specialized to the dreamer and thus to understand the metaphor it is necessary to elicit the input of the dreamer; and emotions guide the metaphorical imagery of the dream (e.g., Freud, 1900; Jung, 1948a; Hall, 1953; Lakoff, 1993; Hartmann, 1996a, 1999a,b; Kunzendorf, 2007).

#### Evidence of Dream Metaphors

To take an example from research, in an interview study we discussed with participants many aspects of dreams that were metaphors for their waking life, such as one participant’s dream of the ‘Starship Enterprise’ taking off from Earth representing her new entrepreneurial career ‘taking off,’ and another participant who dreamt of a demon being released as representing the release of his own ‘inner demon’ (Malinowski et al., 2014). These dreams conformed to the three agreed elements of dream metaphors above: they concretely pictured something abstract from waking life; they were about emotional aspects of their life; and it was necessary to talk in detail with the dreamer about what was going on currently in their life in order to understand the metaphor. Among some other of the more convincing dream metaphor anecdotes in dream research are dreams that clearly figuratively reference some waking-life intrusion during sleep, such as the snake coiling round a participant’s leg in their dream while a pressure cuff was being inflated around their leg (Nielsen et al., 1993), and the participant who dreamt of a bee making a nest in her hair while she slept with EEG electrodes stuck in her hair (Strauch and Meier, 1996).

In addition to these, studies that make use of inductive qualitative methods and follow these up with quantitative methods (blind content analysis) can demonstrate metaphors in dreams. Bulkeley and Bulkeley (2005) noticed that among a group of individuals diagnosed with terminal illnesses dream content often referred to a journey, which the authors inferred was a metaphorical representation of the journey toward death. Some years later in an unrelated study, Bulkeley (2012) found while blind coding a series of dreams that there were an unusual number of transport dreams, and from this and other clues estimated that they came from a similar group of people; the dreams were indeed from a group of older palliative care patients.

#### Functions of Metaphor Generation: Novelty

Metaphor theorists have considered the various ways in which metaphors work: for example, they may simply emphasize the similarity between two things; or, alternatively, they may enable something *new* to be discovered from the comparison (Tourangeau and Sternberg, 1982). Tourangeau and Rips (1991) provided evidence for the latter: metaphor creates an *emergent* feature from two previously unrelated things, rather than simply emphasizing their similarities. Similarly Lakoff and Johnson (1980), who instigated the current understanding of the ubiquity of metaphor in our conceptualization of ourselves and our world, discussed non-conventional metaphors, ones that are not already in existence and common usage, and noted that they are ‘imaginative,’ and ‘creative,’ and can provide new understandings. This is perceptible in dream metaphors: for example, the condensation into one character of a dreamer’s father and male lover, which enables the dreamer to perceive something about the lover that previously they did not (Hartmann, 1996a).

Kuiken (1999) similarly argued that the unidirectional method of interpreting dream metaphors (‘A’ in the dream represents ‘B’ in waking life) is erroneous, and that dreams should be interpreted rather as a composite image, in which A says something about B, and B also says something about A. In this argument also there is an emphasis on novelty, on the combination of two loosely related concepts to produce something new that says something about them both. Indeed, it was found in one study that metaphors created from dream imagery were more novel than those generated from waking fantasy imagery, supporting the novelty-producing concept of dream metaphors (Kuiken and Smith, 1991).

#### Metaphors and Embodied Cognition

The idea of dreams concretely picturing something abstract from waking life does not, however, necessitate that the dream is non-literal, like a spaceship taking off representing a career taking off. Sometimes the concrete representation may be exactly literal, but the way in which it turns the abstract into the concrete is by embodying the thought and making it exist on a physical (albeit imagined) plane. As Hall (1953) wrote, dreams make the conceptual perceptual; and that percept might be a metaphor, but equally it might not be. In their book, Hall and Nordby (1972) discuss the difference between dreams that are ‘denotive,’ that is, the dream imagery stands directly in for its referent, and dreams that are ‘metaphorical,’ in which the dream imagery stands in for something less obvious, and may express complex, even contradictory ideas. Denotive dreams do not require any kind of ‘decoding’ to understand their waking life referent, whereas metaphorical dreams do. Following the logic of the dream and metaphor theorists cited above, it is the metaphorical dreams rather than the denotive dreams that facilitate the making of new connections, the generation of novel ideas. However, it is likely that dreams do not exist in this binary way, denotive versus metaphorical, but that this would be better conceptualized as a continuum. This idea that dreams are embodied simulations of thoughts has been championed by many researchers, such as Domhoff (2011b). In one study he shows how the dreams of a widower about his wife concretely picture his emotional journey after her death (Domhoff, 2007).

Lakoff (1993) notes that metaphors are imagistic – they use imagery from the physical world to conceptualize its more abstract parts – but when used in language they are only pictorial in theory. We do not picture the mental image of someone’s feet being cold when we talk about having “cold feet” before a wedding. However, when dreams use metaphors, they *are* imagistic; dreams are visual, but even more than this they are experiential; they are felt, bodily experiences. Many dream theorists have highlighted the experiential aspect of dreaming, such as Jung, Rogers, and Gendlin, as discussed in a recent article by Ellis (2014). In her work, Ellis shows how re-entering and bodily experiencing a dream can enable the dreamwork to move forward, perhaps by empowering the dreamer to “manipulate their internal imagery system.” There are parallels here between this Focus-Oriented Dreamwork (FOD) and Gackenbach’s findings that high-end gamers seem to have a defense mechanism in their dreams whereby when they have dreams of being attacked they are able to respond in kind because they have rehearsed this behavior in waking life (Gackenbach et al., 2011, 2013). What FOD clients and high-end gamers have in common is that they have rehearsed their adaptive behaviors during wakefulness in lived, bodily, immersive experiences, which then translate into their dream behaviors. Thus, the embodied, lived experience of the imagination is crucial.

#### Functions of Metaphor Generation: Assimilation

Through dreaming embodied metaphors, which are guided by waking emotions, the dreamer is able to assimilate current waking experiences into their wider memory system, and into their mind as a whole. This is the assimilation process: not only are the experiences reactivated in a memory consolidation fashion, but they are lived in the body through imagination and in this way assimilated into information already stored in the brain. This assimilation process is mostly clearly perceived in the research conducted with PTSD patients as discussed earlier. That is, PTSD involves emotional trauma, a general stunting of imagination, and thereby the literal replaying of that trauma during dreaming; recovery from PTSD involves a gradual move into metaphoricalness in dreaming. Dreamwork such as IRT and FOD involves using imagination to change the PTSD nightmares.

Hartmann (1996a, 1999a,b) suggested that dreams are all “explanatory metaphors” for some waking-life emotion, and that this is most obvious in extreme cases such as PTSD and nightmares; ‘normal’ dreams also do this but it is much harder to discern the underlying cause. He also showed that daydreams become *more* metaphorical the more emotional they are, becoming like night dreams (Hartmann et al., 2002–2003). This may at first glance seem at odds with the data that show that dreams become more metaphorical the more adaptation occurs after a trauma in individuals with PTSD; but if the latter is viewed as the extreme end of the spectrum, as a malfunction of the process, it may be speculated that metaphoricalness and emotionality increase together up to a point, but if the emotion is so intense that it becomes traumatic then the ability to produce metaphor fails and is lost, because PTSD stunts imagination. Indeed, we found a large positive correlation between the self-reported emotional intensity and metaphoricalness of late-night dreams (*r* = 0.50), and a medium one in early night dreams (*r* = 0.35; Malinowski and Horton, 2014b). If we interpret these findings in line with the emotion assimilation theory, emotion and metaphor increase together because metaphor generation is a tool for imaginatively thinking about important experiences, and emotion denotes importance. Kunzendorf (2007) noted that one of his participants, who intensified the emotion in a reinterpretation of her dream, in so doing gained insight into it. The intensification of the emotion led to insight. The relationship between metaphor and insight is the focus of the next section.

#### Functions of Metaphor Generation: Insight

Dreamwork often relies on the concept that dreams enable us to find out something about ourselves that we don’t already know (Ellis, 2013); this makes sense in light of the evidence that dreams can be metaphorical, and that metaphors generate novel thoughts. This view, however, is in stark contrast to the beliefs of some researchers who claim that dreams *never* tell us anything we don’t already know (e.g., Hobson in his dialog with Schredl: Hobson and Schredl, 2011). The difference perhaps rests on differing notions of what dreams ‘are’: the self-organization of random neural firings in the brain (e.g., Kahn and Hobson, 1993), or the self-organization of meaningful, purposeful, patterned firings of pieces of information with a tendency toward emotionality that the dreamer is not aware of but that would be useful to be aware of. Thus it is necessary to define what is meant when we say “something we don’t already know.”

Creative insight is defined as the association of elements of information already stored in the mind into new configurations that are in some way useful (Cai et al., 2009). In Cai et al.’s (2009) study, the ability to discover associated words in the Remote Associations Test was significantly enhanced by REM (but not NREM) sleep, when the participants had (unknowingly) been primed on the answers before sleep. Thus REM sleep facilitated this associativity ability during subsequent wakefulness. Sleep processes – and we would argue, the concomitant subjective experience of dreaming – enabled the hyperassociativity of the information that was already stored to be used in a creative and useful way. Similarly, working with dreams has been shown to generate insight into the self (Edwards et al., 2013, 2015), and more insight is obtained when participants are asked to make associations between the dream images and waking life than when simply richly describing the dream images (or a combination of the two: Hill et al., 1998). Perhaps then the idea of dreams telling us “something we don’t already know” should be restated as “something we are not aware of knowing,” in which ‘knowing’ may constitute formerly disparate pieces of information that require reconfiguring to become useful. Dreamwork facilitates obtaining insight into these non-conscious thoughts, perhaps through the unraveling of dream metaphors, which, as discussed, are potentially particularly useful for making new discoveries. However, the concept that ‘decoding’ dream metaphors can enable the generation of insight currently lacks empirical evidence, so this idea is very much speculative at present.

Thus we believe that, despite the difficulties in objectively demonstrating the existence of metaphor in dreams, there is evidence enough to make the assertion that abstract waking-life thoughts appear in dreams in concrete ways; sometimes this concrete representation is ‘denotive,’ that is, a literal representation, and other times it is ‘metaphorical,’ that is, a non-literal representation (and variations in between these two states). These metaphorical representations are guided by emotions, enable the assimilation of emotions, and have the potential to generate novel insights, the uncovering of which can be facilitated by dreamwork. PTSD dreams are an extreme or a malfunction of this process, and so are not metaphorical at all, until the recovery process begins, which may be offset by ‘kick-starting’ the imagination through types of dreamwork specifically designed for this purpose.

### Hyperassociativity

The intense connectivity between loosely associated memories (‘hyperassociativity’) that may be behind insight and creativity during sleep is the second kind of imaginative activity that occurs particularly during sleep/dreaming, and it is to this that we now turn. There is wide agreement among researchers that dreaming, unlike focused waking thought, is hyperconnective, fluid, and flexible (Antrobus, 1993; Globus, 1993; Montangero, 1993; Hartmann, 1996a; Cai et al., 2009; Levin and Nielsen, 2009; Walker, 2009; Walker and Stickgold, 2010; Cartwright, 2011; Llewellyn, 2013). It has something in common with daydreaming and other loose modes of thinking during wakefulness but is more extreme than them (Hartmann, 2010; Fox et al., 2013), and also with the ‘default network’ of the waking brain (Domhoff, 2011a). Many researchers agree that the dream is always a novel creation, and is often bizarre because of its intense interconnectivity (Montangero, 1993; Hartmann, 1996a; Stickgold and Walker, 2004; Cartwright, 2011).

#### Evidence of Hyperassociativity in Dream Content

In Stickgold and Walker’s (2013) triage theory, it was suggested that once memories have been selected for incorporation, two main processes then occur during sleep: item integration, in which newly learnt memory representations are assimilated into pre-existing schemas; and multi-item generalization, in which new items are combined into a *new* schema. It is this latter process that is particularly of relevance here: this multi-item generalization stage may be reflected in dreams as hyperassociativity. At this subjective, dream level, hyperassociativity is reflected in at least three ways. First, dreams do not replay waking-life experiences ‘intact’ but rather incorporate them in fragmentary ways, with disparate elements of waking life appearing in one dream narrative (Fosse et al., 2003; Malinowski and Horton, 2014c; Horton and Malinowski, 2015). One study found that 81% of dreams contained abrupt and complete changes of dream events within the dream narrative (Montangero, 2012). During this hyperassociative process, episodic memories are “unbound” or “disconnected” (Payne, 2010, p. 122). The second way in which dreams reflect hyperassociativity is in their bizarreness: disparate, disjointed elements of memory join (associate) together with imagination to form bizarre narratives (e.g., Revonsuo and Tarkko, 2002; Levin and Nielsen, 2007; Montangero, 2012).

The third potential way in which dreams are hyperassociative is in time variance: dreams interweave elements of the past, the present, and the imagined or anticipated future. For instance, Cartwright et al. (1984) found that depressed divorcees had dreams that were often stuck in the past, whereas non-depressed divorcees’ dreams exhibited more time variance. Given that many of the theories discussed so far have proposed a mechanism whereby current information is interweaved during sleep with pre-existing information, including ‘gist’ memories as well as more specific memories (e.g., Breger, 1967; Walker and Stickgold, 2010), it makes sense that dream content that is related to a heightened ability to assimilate this information (and therefore cope with it) exhibits time variance. At the other end of the spectrum are individuals whose ability to assimilate information has been enormously reduced, such as PTSD patients. One of the effects of PTSD symptomatology is the appearance of more general, and less specific, autobiographical memories (Moore and Zoellner, 2007), and this ‘overgenerality’ has been found to apply also when imagining the future (Brown et al., 2013). Additionally, PTSD patients who were manipulated to believe they were high in self-efficacy were found to generate memories and imagined future events with greater specificity than those who believed they were low in self-efficacy (Brown et al., 2012). This, and the evidence from Cartwright’s study in the previous paragraph, indicate that time variance, as one mode of hyperassociativity, is crucial to memory integration and emotion assimilation.

Several studies evidence that dreams respond associatively to waking-life stimuli. Davidson and Lynch (2011) found that, after exposure to a video depicting the events of 9/11, participants had more dreams of material that was literally, closely and loosely related to material, demonstrating the hyperassociative pathways active during dreaming. Similarly, naturalistic post-9/11 studies generally found little evidence for direct incorporations of the event into dreams, but did find evidence for associated images, themes, and emotions (e.g., Hartmann and Basile, 2003; Hartmann and Brezler, 2008). Domhoff (1993), in commenting on the resurgence of traumatic dreaming of Vietnam veterans during much later times of unrelated stress (such as marital difficulties), suggested that the dreams could be metaphorical representations of the present stress; conversely, perhaps the current stress simply opened up associative pathways. Similarly, post-traumatic nightmares can be triggered by a new life event or even a television image up to 40 years after the traumatic experience (Schreuder et al., 1998; Schreuder et al., 2000). Given that it is unlikely that a traumatic nightmare is a metaphorical representation of a television image, it seems more likely that associative pathways to the traumatic experience were re-opened by the associated image.

The fact that dreams involve elements unknown to the dreamer (such as characters or activities that the dreamer has not had direct experience of in waking life) is not evidence against the notion of these being distant memory associations, because memory consolidation does not involve merely strengthening memories for literal recall, but also extraction of the gist of the memory (Wamsley, 2014). Indeed, we have found that ‘generic’ elements occur frequently in dreams, such that people dream of recognizable ‘types’ – which come from an amalgamation of a variety of specific waking-life memories, but not any one particular memory – which are akin to ‘extras’ in a film (Malinowski et al., 2014). For example, one participant dreamt of a generic bookshop and a generic bookshop assistant, which comes from a combination of various experiences of bookshops and bookshop assistants, but without being any particular place or person from memory. Strauch and Meier (1996) call such generic elements the “building blocks” of dreams.

#### Functions of Hyperassociativity: Assimilation

Many of the researchers cited in Section “Emotion-Processing Theories of Dreaming” who postulated emotion-processing theories of dreaming hypothesize that this process occurs via hyperassociativity (e.g., Breger, 1967; Hartmann, 1996a; Levin and Nielsen, 2007; Cartwright, 2011). Breger talked about the dream providing a context in which mnemonic information is more freely available to be accessed than during waking; and moreover, it is available to be accessed in a fluid, associative manner. This “creative opening up of the memory systems” (p. 25) is what enables integration of new with old memories. Llewellyn (2013) postulates that recent episodic experiences are hippocampal-dependent and are assimilated into long-term, more stable emotional memory networks during dreaming. Thus dreaming provides a context in which experiences can be *elaborated*, activating relational memories. Hartmann (1996a) drew similar conclusions, envisaging the brain as an interconnected network of units, and suggesting that the flow of information between these units during waking (especially during directed thought) is linear, and this is most functional when we are in pursuit of a goal. However, during dreaming, the flow of information is non-linear, and may spread laterally into units that are more loosely connected together; this process is guided by emotion. Thus, PTSD dreams are a dysfunction of this process, and are not hyperconnective but are still guided by emotion, whereas ‘normal’ dreams following trauma are hyperconnective *and* guided by the emotion. As the dreams move from traumatic to normal, they become more hyperconnective, and thus the original emotion becomes harder to determine. This process contextualizes the current emotional concern in a different (novel) perspective, and in situations that have been experienced before. In this view, new material is interweaved with older material to form new creations, in order to adapt to experiences.

#### Functions of Hyperassociativity: Insight

These processes are envisioned to occur primarily during REM sleep, the stage of sleep most conducive to hyperassociative thinking. In addition to Cai et al.’s (2009) findings (see Functions of Metaphor Generation: Insight), a number of other studies evidence that REM sleep facilitates hyperassociative thinking, which may lead to insight. Stickgold et al. (1999) found that weak priming (e.g., ‘crime’ with ‘gun’) exceeds strong priming (e.g., ‘hot’ with ‘cold’) after REM sleep, suggesting again that hyperassociative thinking is occurring during REM sleep. Walker et al. (2002) found that participants awoken from REM sleep were better at solving anagrams than those awoken from NREM sleep, again demonstrating that REM sleep is conducive to associative modes of cognition. Recently, Carr and Nielsen (2015) have shown that a REM-sleep group of participants scored higher than NREM or wake groups on primed emotional stimuli using the associational breadth method. Some behavioral paradigms have demonstrated the overall role of sleep, as opposed to wake, in enhancing insight into associations between learned categories (Ellenbogen et al., 2007). Taking the evidence together, memory researchers posit a complex picture of the brain during sleep; far from mere consolidation (strengthening) of memories during sleep, the brain is undergoing a whole host of memory *transformation* processes, including assimilation and integration, abstraction and generalization, selective extraction of useful information, and generating insights from the information stored (Payne, 2010; Walker and Stickgold, 2010; Llewellyn, 2013).

In Section “Metaphors and Embodied Cognition” and “Functions of Metaphor Generation: Assimilation” it was postulated that metaphor generation may be instrumental in the novelty- and insight-producing abilities of dreaming. Similarly hyperassociativity has the potential to produce novelty and new insights. Kuiken and Smith (1991) suggest that new meanings are reached in dreams during ‘visual reorientations’: things changing appearance, sudden scene shifts, the dream self suddenly realizing something, etc.; things that often typically make up the categories in content analysis of dream bizarreness (e.g., Revonsuo and Salmivalli, 1995). This bears relation to White’s (2014) findings on counterfactual thinking in dreams: he found that when the dream self attempts to solve a problem within a dream, they usually fail. However, when new information just ‘appears’ in the dream – which can be in the form of a character suddenly appearing, or new knowledge suddenly appearing in the dream self’s mind, or any other sudden change – this usually helps to move the dream self forward.

Both Kuiken and Smith’s (1991) and White’s (2014) research can be interpreted as further evidence for hyperassociativity in dreams. The sleeping mind already has the information required to move the dream forward or come to novel understandings, and this information seems to just ‘appear’ in the dream because an associative network has suddenly been activated during sleep; the subjective experience of this is a sudden appearance of a person or a thought. The importance of the sudden appearance of something new in a dream is also echoed by Ellis (personal communication) in relation to PTSD dreams: “[a PTSD nightmare] repeats and repeats until something new or different happens that can move the process forward as intended.” These sudden openings to associated information stored somewhere in the brain may be behind the insight that is generated during REM sleep. Research is needed to find out what the subjective experience is behind such insights, but it may be that it is these sudden changes in dreams, reflecting sudden ‘firings’ of associative networks.

#### The Relationship between Hyperassociativity and Metaphor

Closely related to the idea of hyperassociativity and creativity are the ideas of connectionism and self-organization. Like Hartmann (1996a), dream connectionist theorists such as Globus (1993) discuss the mind as a network of interconnected ‘nodes’ (neurons) that operates under a parallel processing system, spontaneously reorganizing the entire make-up of the system in response to input. Relatedly, Antrobus (1993) argues that memories for things like attributes, objects, and events are stored as a distributed pattern, which are connected by conceptual similarities. When a unit has a similarity another unit, those units have an active, connected relationship (such as ‘leg’ and ‘flexible joint’), but when they are incompatible with another unit, they have an inhibited, disconnected relationship (such as ‘leg’ and ‘rigid’). Units with such shared conceptual features may be more likely to be connected during dreaming, which enables dreams to be representative or “metaphoric” (p. 81) in quality.

In this view, associativity and metaphor go hand-in-hand during sleep/dreaming; thus there should be a relationship between metaphor and bizarreness, if bizarreness is a measureable marker of hyperassociativity. We found a very large positive correlation between self-reported metaphoricalness and bizarreness in participants’ late-night dreams (*r* = 0.72) and a large positive correlation in early night dreams (*r* = 0.51; Malinowski and Horton, 2014b). Similarly, since we argue that the emotion-assimilation function of dreaming is predicated on imaginational abilities, and that dream bizarreness (for hyperassociativity) is one of these abilities, it would be expected that bizarreness and emotional intensity are also positively correlated. There were medium-to-strong positive correlations between emotional intensity and bizarreness in both late-night dreams (*r* = 0.46) and early night dreams (*r* = 0.38). Future research should look into whether these correlations are particular to positive or negative emotions, to throw further light onto whether it is particularly negative emotions that are required to be assimilated via the imaginational properties of dreaming, or whether positive emotions are equally dreamt of in this way.

#### Temporal Effects of Hyperassociativity

Hyperassociativity may also be evident as a long-term, iterative process, as has been discussed, and illustrates how dream incorporation and adaptation occur after the new emotional experience has been activated, both across multiple nights and within a single night. Blagrove et al. (2011a,b) have replicated the findings of Nielsen and Powell (1992) regarding the dream-lag effect: that is, waking-life experiences tend to appear in dreams the night immediately after they have occurred, and then not again until 5–7 days later; but on second appearance, the dream incorporates the experience in a more abstract way. This could be evidence for the experience being interweaved into the wider memory system, i.e., connections being made between the new information, established information, and imagination. More recently, van Rijn et al. (2015) have found that the dream-lag effect only exists for personally significant events, and not major daily activities or major concerns. This is important because ‘personally significant event’ implies emotional in both positive and negative ways, whereas ‘major daily activities’ implies everyday, neutral experiences, and ‘major concerns’ implies specifically negative thoughts and experiences.

Within a single night, several stage of sleep and time of night effects have been found in dreams: REM dreams and those toward the end of the night tend to become longer, more vivid, more emotional, and more bizarre (Hobson et al., 2000; Wamsley et al., 2007; Malinowski and Horton, 2014b), although REM and NREM dreams become more similar as the night progresses (Nir and Tononi, 2010). Similarly Wamsley et al. (2010) found that incorporation of waking life experiences increased in abstraction with duration of sleep. Cartwright also found (Section “Cartwright: The Emotion Regulation Function of Dreaming”) that dreamers who achieve emotional adaptation tend to start out dreaming in negative tones in the early night and end up in positive tones in the late night. Thus a picture begins to build for dreaming across the night, where bizarreness, emotional intensity, and emotional valence change as the night goes on. More research is required to get a fuller picture of these changes, considering metaphor also (Malinowski and Horton, unpublished manuscript).

Similarly, time of night influences memory consolidation during sleep, such that episodic memories tend to be preferentially consolidated during NREM sleep in the earlier portion of the night, and procedural and emotional memories during REM sleep in the later portion of the night (Plihal and Born, 1997; Payne, 2010). Such evidence has led some researchers to propose a memory consolidation theory of sleep whereby slow wave sleep (SWS) initially consolidates recent episodic material (such as from the previous day), and REM sleep integrates and abstracts it (e.g., Giudatta et al., 1995, see also Llewellyn, 2013). Such theorizing is in line with the dream research findings, since early night (SWS) dreams tend to reflect current concerns, and later night (often REM) dreams tend to be more bizarre, which may mean they are more hyperassociative, as above. This in turn may reflect the subjective experience of the memory integration function of REM sleep. Whilst we recognize that dreams from late-night sleep are typically more bizarre than those elicited from early night sleep, we are not in a position to postulate that the emotion assimilation processes are specifically REM-dependent. Rather, they may require both non-REM and REM processing (Ellenbogen et al., 2007; Cairney et al., 2014), or be a function of time spent asleep.

At the chemical level, these time of night changes in hyperassociativity may be understood as a function of the circadian cycle of the stress hormone cortisol (Payne, 2010). Traumatic experiences, which are accompanied by the release of cortisol, are often fragmented and lack coherence, with little episodic or contextual information retained. Conversely, emotional material is ‘stamped in’; the resultant effects are either disjointed memory fragments, or memories that undergo “narrative smoothing” (p. 117), the latter of which is similar to the synthesis of memory fragments into a narrative in dreams. Thus, since levels of cortisol increase across the night and peak in the late morning, so dreaming becomes more fragmented and bizarre across the night, peaking in the late morning.

Hyperassociativity during sleep/dreaming, then, appears to index one of the stages of memory consolidation during sleep: that is, the stage during which memories are integrated, which happens during late-night, predominantly REM, sleep. During this stage of the memory consolidation process, recent memories are activated and associated with other information already stored in the memory system. There is widespread evidence for hyperassociativity occurring during sleep, such as the insight generated following REM sleep compared to NREM and wakefulness; likewise there is widespread evidence for hyperassociativity during dreaming, such as time variance in dreams, the activation of associated memories following exposure to stimuli, and the fragmentariness and bizarreness of some, particularly late-night, dreams. Several researchers conceptualize the mind in this state as a series of interconnected nodes, activating in parallel rather than the more sequential, or linear, mode of thought common to focused waking cognition. Hyperassociativity, like metaphor, has the potential to create novel insights. It increases over time, both within a single night (bizarreness increases toward the end of the night and episodic replayings of memories decrease; this may be seen at the chemical level in increases in cortisol), and across many nights (abstraction increases with repeated incorporations, as evidenced by dream-lag studies).

## Suggestions for Future Directions

As we have emphasized throughout, theories of emotional processing during sleep could benefit from exploring dream content, and theories of dreaming could benefit from considering the behavioral and neurological profile of the sleeping brain. In practice empirical studies from the sleep lab need to be followed up with dream studies. Dreams can be studied as transparent reflections of sleep-dependent memory processes (Wamsley, 2014), provided they are sampled under ideal conditions (Windt, 2013), taking heed from the systematic and rigorous methodologies typifying sleep science (Kahan and Horton, 2012). More specifically, there are some aspects of our proposed emotion assimilation theory that would benefit from greater exploration. The time-course of the emotion assimilation processes is as yet understudied, with the domains of sleep science and dreaming sometimes focusing on different levels of enquiry, with sleep-labs typically exploring shorter time frames, from naps (e.g., Payne et al., 2015) to around 24 h periods (Ellenbogen et al., 2007) compared to some longer-term dream studies (e.g., Cartwright’s divorcee studies, Section “Cartwright: The Emotion Regulation Function of Dreaming.”) Similarly the role of REM sleep, in particular, requires further consideration in the selection of emotional information, and its subsequent consolidation.

The differential effects of positive and negative emotions in the consolidation and assimilation process should also be parsed out. Kensinger (2009) has reviewed the effects of emotion on (non-sleep-dependent) memory processes, and found that while emotion has a beneficial effect for memory whether it is positive or negative, the benefits are different: positively valenced memories are more likely to be remembered in terms of the gist of the memory, whereas negatively valenced memories are more likely to recalled in specific detail. That is, she found a link between negative emotion and sensory processing on the one hand, and positive emotion and conceptual processing on the other. These effects should be investigated in terms of the effects of differently valenced memories and sleep, and the phenomenology of these effects during sleep by investigating dream content.

Dream science can be particularly insightful in terms of the study of metaphors and associations between memory elements to reflect assimilation. It has been over 10 years since Domhoff’s (2003) suggestion that inductive qualitative methods, followed up with objective quantitative methods, are used to study dream metaphors in depth, but few researchers have broached this topic yet. More research is needed to investigate the conscious experiences of assimilation that occur during sleep in dreams, and dream metaphor, hyperassociativity, and bizarreness may provide starting points. Similarly, dream content may be fruitful in the study of other memory processes during sleep, such as gist extraction and insight generation.

Finally, links between dream content and more general memory behaviors are needed, such as exploring dream content from periods of successful consolidation (versus less successful consolidation) and, where possible, identifying specific links between dream content and enhanced recall of those memories activated in dreams (as in Horton, 2012). More generally, exploring the content of dreams taken from sleep that enhances creativity, insight or problem solving may also shed light on the sleep-dependent processes that give rise to such successful cognitive functioning.

## Conclusion

We have proposed (i) that emotion acts as a marker for information to be selectively processed during sleep, including consolidation into long term memory structures and integration into pre-existing memory networks, (ii) that dreaming reflects these processes of emotion assimilation, and (iii) that the associations between memory fragments activated during sleep give rise to measureable elements of dream metaphor and hyperassociativity (e.g., time variance and bizarreness). In turn, these elements indicate the time-course of emotion assimilation, with greater bizarreness signifying greater distance from the original emotionality of the experience, in turn indicating more advanced assimilation.

These processes directly compliment the proposed model of autobiographical memory functioning in the sleeping brain (Horton and Malinowski, 2015) which describes how waking-life elements are broken down into fragments, subsequently re-bound together via hyperassociativity during sleep, and manifest in bizarre experiences of dreams and the metaphorical representation of salient and emotional waking life experiences. In our emotion assimilation theory we emphasize how emotion during sleep can function to improve the retention and retrievability of salient experiences so we can make use of them better in the future. Taken together, experiences and information from waking life can be broken down for oﬄine filing during sleep, and activated as part of that process. Associated emotionality of those memory fragments leads to heightened activation, in turn resulting in further hyperassociativity, and as such a stronger memory consolidation process ensues.

Currently there is not enough evidence to make the claim that experiencing dreaming contributes to emotional assimilation directly; rather, dreaming can be taken to reflect the activation of memories whilst the brain’s sensitivity to external stimuli is lessened (Murkar et al., 2014). Dreaming provides a methodological tool for exploring cognitive processing during sleep, though at this stage we are not able to measure the additional value of dreaming versus not-dreaming to these processes. (See Kahan and Horton, 2012, for a brief review of dreaming as a methodological tool.) We assume that dreaming occurs in the vast majority of individuals each night during sleep, though being able to remember one’s dreams is independent of the sleeping brain’s ability to assimilate emotional material into pre-existing networks. Further, although we propose that sleep may serve the function of assimilating emotional information into a stable, organized network of personal and semantic information, we make no claims about this being sleep’s only, or even main, function. Similarly, we recognize that sleep has evolved and its primary functions likely changed, with functions such as emotion assimilation being a consequence of a rapidly evolving and stimulated human brain in everyday contexts.

## Conflict of Interest Statement

The authors declare that the research was conducted in the absence of any commercial or financial relationships that could be construed as a potential conflict of interest.
